# Neonatal transport in resource restricted settings: a simple clinical score at arrival and its role in predicting mortality

**DOI:** 10.1186/1865-1380-7-S1-P2

**Published:** 2014-07-25

**Authors:** Deepak Rathod, Bethou Adhisivam, Vishnu Bhat

**Affiliations:** 1 Department of Paediatrics, Jawaharlal Institute of Postgraduate Medical Education and Research, Puducherry, India

## Objectives

To describe the indications and mode of transport of extramural neonates to JIPMER NICU.

To qualify the condition of neonates at arrival using a simple clinical score.

To analyze the effect of this clinical score on outcome.

## Methods

Setting: JIPMER, a tertiary care teaching hospital, south India.

Number of subjects: extramural neonates (<28 days) transported to JIPMER NICU (n=303).

Study design: descriptive study.

Transport and clinical details were recorded at arrival to NICU and all neonates were evaluated using a simple clinical score - sick neonate score (SNS) as described below in Table 1.

**Table 1:** 

Variable	Score		
	
	0	1	2
Respiratory effort	Apnea or Grunting	Tachypnea (>60/min) with or without retractions.	Normal (40-60/min)

Heart rate	Bradycardia asystole	Tachycardia ( >160/min)	Normal (100-160/min)

Mean blood pressure (mmHg)	<30	30-39	>39

Axillary temperature (^0^C)	<36	36-36.5	36.5-37.5

Capillary filling time (secs)	>5	3-5	<3

Random blood sugar (mg/dl)	<40	40-60	>60

SpO_2_ (in room air)	<85%	85-92%	>92%

All neonates were followed up until discharge or expiry. To determine the association of clinical factors with outcome Chi-square and Fisher exact test were used. All statistical analyses were carried out at 5% level of significance and p value <0.05 was considered significant. To determine the cutoff value for the clinical score (SNS), receiver operated curve was plotted.

## Results

Most of the neonates were transported by private ambulance (36%), followed by taxi (29%), bus (15%), 108 service (11%), two-wheeler (6%) and auto (3%). The indications of transport were sepsis (30.7%), HIE (17.5%) and respiratory distress (15.2%). Among 60 expired neonates 76% were hypothermic. SNS ≤8 predicted mortality. All the components of SNS significantly correlated with outcome (i.e) lower the score, poorer the outcome. Neonates transported by 108 services and private ambulances had better SNS and outcome compared to neonates transported by other modes of transport .

## Limitations

No pretransport score was available.

## Conclusion

Neonatal transport in the region is not satisfactory and SNS is a useful clinical score for predicting mortality.

